# Advancing Cancer Therapy with Present and Emerging Immuno-Oncology Approaches

**DOI:** 10.3389/fonc.2017.00064

**Published:** 2017-04-18

**Authors:** Jeff Kamta, Maher Chaar, Anusha Ande, Deborah A. Altomare, Sihem Ait-Oudhia

**Affiliations:** ^1^Center for Pharmacometrics and Systems Pharmacology, Department of Pharmaceutics, College of Pharmacy, University of Florida, Orlando, FL, USA; ^2^Burnett School of Biomedical Sciences, College of Medicine, University of Central Florida, Orlando, FL, USA

**Keywords:** cancer immunotherapy, checkpoint inhibitors, cytotoxic T-lymphocyte-associated protein 4, programmed cell death protein 1 and PD-L1, adoptive cell transfer, oncolytic viruses, bispecific T-cell engagers, cancer vaccines

## Abstract

Immuno-oncology (I-O) is a young and growing field on the frontier of cancer therapy. Contrary to cancer therapies that directly target malignant cells, I-O therapies stimulate the body’s immune system to target and attack the tumor, which is otherwise invisible to, or inhibiting the immune response. To this end, several methods have been developed: First, passive therapies that enable T-cells to fight the tumor without direct manipulation, typically through binding and modifying the intracellular signaling of surface receptors. Checkpoint inhibitors, perhaps the most well known of I-O therapies; are an example of such. These are monoclonal antibodies that block binding of the tumor cell at receptors that inactivate the T-cell. A variety of small molecules can achieve the same effect by affecting metabolic or signaling pathways to boost the immune response or prevent its attenuation. Drugs originally formulated for unrelated disease states are now being used to treat cancer under the I-O approach. Second, active therapies which often involve direct manipulations that occur *in vitro* and once introduced to the patient will directly attack the tumor. Adoptive cell transfer is the oldest of these methods. It involves the removal of T-cells from the body, which are then expanded and genetically modified for specificity toward tumor-associated antigens (TAAs), and then reintroduced to the patient. A similar approach is taken with cancer vaccines, where TAAs are identified and reintroduced with adjuvants to stimulate an immune response, sometimes in the context of antigen-presenting cells or viral vectors. Oncolytic viruses are genetically modified natural viruses for selectivity toward tumor cells. The resulting cytotoxicity has the potential to elicit an immune response that furthers tumor cell killing. A final active approach is bi-specific T-cell engagers. These modified antibodies act to link a T-cell and tumor cell through surface receptors and thereby forcibly generate immune recognition. The therapies in each of these subfields are all still very new and ongoing clinical trials could provide even further additions. The full therapeutic potential of the aforementioned therapies, alone or in combination, has yet to be realized, but holds great promise for the future of cancer treatment.

## Introduction

Immuno-oncology (I-O) is a young and growing field, the product of the many groundbreaking discoveries in immunology and cancer therapy in the last century. The novelty of this field is due to the historical controversy over whether or not the body’s immune system could even respond to cancer at all. The idea was first proposed by William Coley in 1893, when he observed the remission of cancer in patients who had contracted acute bacterial infections (1); then followed by Paul Ehrlich in 1909, when he suggested that the immune system must have some role in preventing an outbreak of cancer in the body (2). Unfortunately, their theories were opposed by those who did not see a plausible biological explanation, and who were convinced that cancer cells were indistinguishable from healthy cells to the body’s lymphocytes. The breakthrough for modern I-O came in the 1960s, when it was accepted that lymphocytes are constantly eliminating precancerous cells throughout the body in a process called “immune-surveillance,” and do in fact recognize them through tumor-associated antigens (TAAs) (3). This has gradually led to our understanding of cancer today.

As we learn more about the relationship between cancer cell and lymphocyte, therapies continually improve. IL-2 therapy was the first to be approved, based on the finding that the IL-2 cytokine stimulates T-cell production and would therefore enhance its activity against the tumor (4). Hampered by toxicity, the clinical outcomes were not as positive as hoped. Heightened promise came to the field in the form of targeted antibodies, cancer vaccines, and more recently, a shift in focus from targeting the cancer directly to targeting the lymphocytes that have been disabled in the tumor microenvironment. These checkpoint inhibitors re-employ lymphocytes to perform the crucial duties that scientists only recently discovered.

The manipulation of immune checkpoints is the leading edge for the field of I-O. The activation of a lymphocyte, such as a cytotoxic T-cell, in adaptive immunity has been well-characterized and is known to involve the interaction between an antigen-presenting cell (APC) and the T-cell receptor (TCR), and associated coreceptors. Less explored are immune checkpoints, the body’s natural defense against auto-immunity. This involves the binding of receptors on the lymphocyte with associated ligands on the surface of the cancer cell that interfere with activation signals or induce apoptosis. Inhibition occurs almost as quickly as activation and balances the antigenic response of immune cells to avoid an attack on healthy cells. Tumor cells exploit this with an upregulation of inhibitory ligands, leaving them free to grow unchallenged by the immune system.

With many of its greatest leaps coming within the last 30 years, I-O has been touted as an overnight success. The famous six hallmarks of cancer described by Hanahan and Weinberg in their influential 2000 paper was updated in 2010 with four new hallmarks (5), one of which being the ability of a cancer to “be invisible to the body’s immune system.” I-O and its advancements were named the 2013 scientific breakthrough of the year by “Nature” (6). Keeping on the forefront of the fight against cancer now means being aware of advances in I-O.

## Passive Therapy of Biological I-O Treatments

The classical way of treating cancer is by directly targeting the tumor. However, passive therapies facilitate the body’s existing immune response and do not require direct participation of the immune cells.

### Monoclonal Antibodies (mAbs)

Monoclonal antibodies are produced *in vitro* and can be of varying origins, such as murine, chimeric, humanized, and human. These antibodies are specific to a TAA and when administered can attack the tumor cell in various ways. One way is through antibody dependent cell mediated cytotoxicity. This occurs when the therapeutic mAb attaches to a specific surface antigen on the tumor cell and to the Fcγ receptor of the immune cell, usually natural killer (NK) cells or macrophages. Effector cells will then enzymatically destroy the cancer cell. Alternatively, antibodies may activate the complement system, a group of proteins that form a membrane attack complex in response to antibody tagged cells, which is used to perforate the cell membrane and cause death. Antibodies might also be conjugated to a chemotherapeutic or radioactive drug, and used to fight the tumor by facilitating delivery of this drug directly to cancer cells. mAbs have topped biologic sales since 2009 achieving $24.6 billion within the U.S. market (7). Owing to their advantages of high specificity and potency, they can be effectively developed into targeted therapies eliciting high efficacy and low toxicity when compared to small molecule drugs. FDA-approved mAbs reached 52 by the end of 2015 that included naked mAbs as well as antibody drug conjugates. For instance, Trastuzumab (Herceptin–Genentech) is a humanized mAb that acts by targeting HER2 receptor thereby suppressing proliferation and survival of HER2-dependent tumors in HER2 overexpressing breast cancer patients (8). Similarly, Bevacizumab (Avastin–Genentech) which acts by inhibiting VEGF signaling mediated angiogenesis, has been indicated for first-line treatment of various cancers including metastatic colon cancer, non-small cell lung cancer (NSCLC) in conjunction with chemotherapy (9). The following section outlines in detail other classes of mAbs.

#### Cytotoxic T-Lymphocyte-Associated Protein 4 (CTLA4) Inhibitors

**Figure 1:**
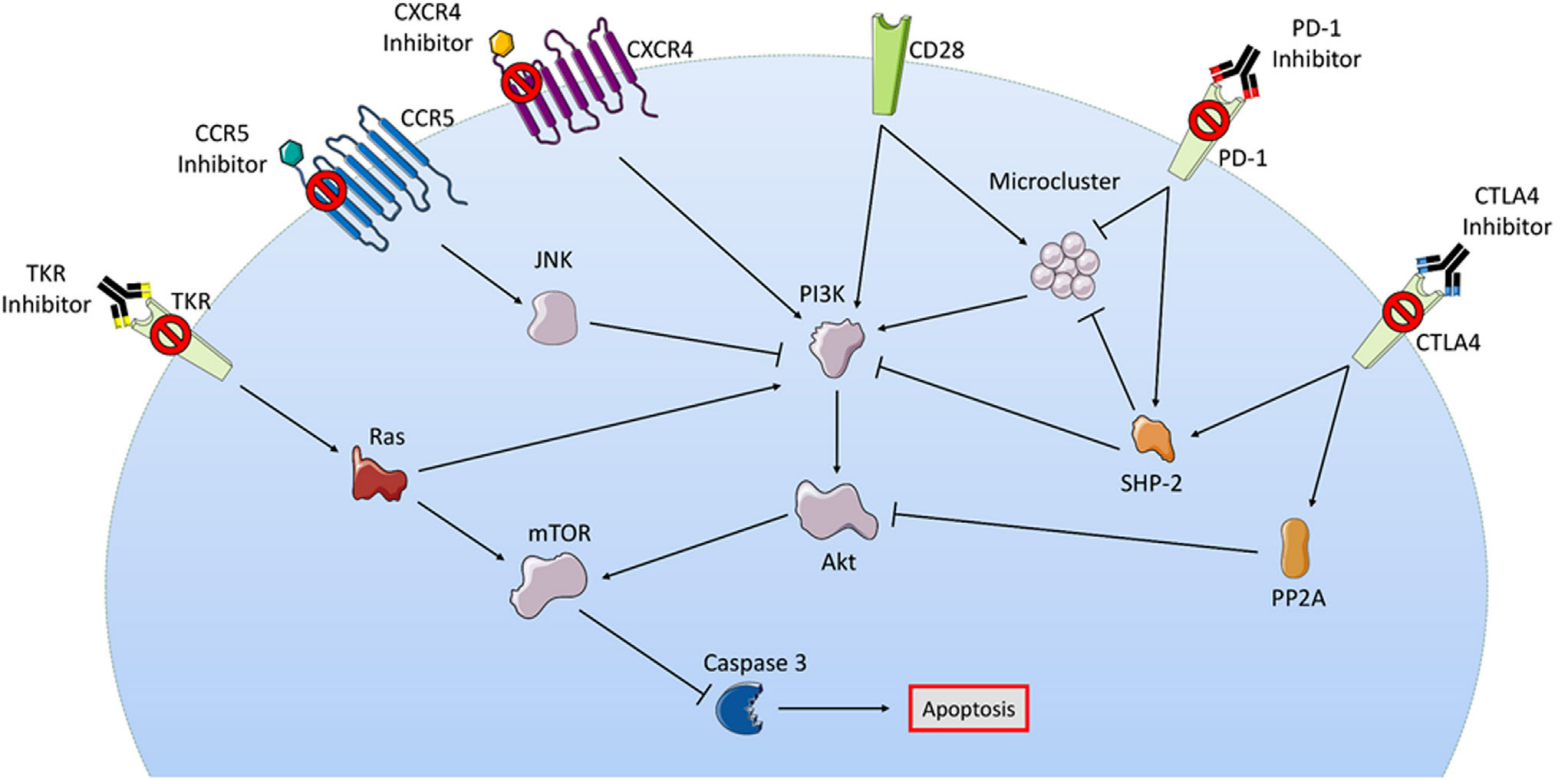
**Although a myriad of intracellular signaling is involved in passive therapies, their pathways show some overlap**. In this figure, the checkpoint receptors PD-1 and CTLA4 are both shown to activate SHP2 leading to inhibition of microcluster signaling molecules, indirectly opposing the actions of activated stimulatory receptor CD28; checkpoint blockade prevents T-cell inhibition in this regard. Small molecule targets such as CXCR5, CCR5, and TKR are all also shown to converge with the checkpoint inhibitor MOA at the PI3K/Akt survival pathway, some through inhibition or some through activation depending on whether these effects are seen in T_reg_ or T_eff_. Intermediaries such as JNK and Ras are seen with CCR5 and TKR, respectively. Downstream of all pathways is the activation or inhibition of caspase-mediated apoptosis. Abbreviations: CCR5, C-C chemokine receptor type 5; CTLA4, cytotoxic T-lymphocyte-associated protein 4; CXCR4, C-X-C chemokine receptor type 4; JNK, c-Jun N-terminal kinase; mTOR, mechanistic target of rapamycin; PD-1, programmed cell death protein 1; PI3K, phosphatidylinositol-3-kinases; PP2A, protein phosphatase 2a; TKR, tyrosine kinase receptor. Some elements adapted from Servier Medical Art (https://creativecommons.org/licenses/by/3.0/legalcode).

Cytotoxic T-lymphocyte-associated protein 4 was the first immune checkpoint to be used as a drug target and promoted the field of I-O. CTLA4 is a receptor expressed on the surface of activated T-effector cells (T_eff_), and T-regulatory cells (T_reg_), and when bound causes inhibition of the T_eff_ and enhancement of T_reg_. Expressed on the surface of T_eff_ cells also is CD28, which is homologous to CTLA4 and functions to stimulate the cell. CD28 and CTLA4, therefore, counteract each other (Figure 1), and also compete for the same two receptors on APCs, namely CD80 and CD86. CTLA4 has higher affinity for both ligands and naturally outcompetes CD28. This functions to temper the immune response and prevent autoimmune reactions (10). However, the overexpression of CTLA4 in various cancers has led to uncontrolled tumor growth. Activation of the T_eff_ cell is mediated through costimulation of both the TCR and CD28. Coligation of both receptors is critical as activation of the TCR alone has been shown to paradoxically result in T-cell anergy, whereas activation of CD28 alone is insufficient to cause T-cell activation (11).

Prior to T-cell activation, CTLA4 remains sequestered intracellularly in endosomes, lysosomes, and the trans Golgi network (TGN). Interaction between the T-cell and APC creates an immune synapse, which prompts upregulation of CTLA4 and its migration to the membrane at the site of the synapse. Vesicular budding from the TGN occurs with the assistance of ADP-ribosylation factor 1 (12) and phospholipase D. CTLA4 is then shuttled to the membrane by a tripartite motif protein (11). Ligation of CTLA4 will lead to phosphorylation of the enzyme phosphoinositide 3-kinase (PI3K), and the recruitment of the phosphatase PP2A intracellularly, thereby inhibiting the PI3K and Akt survival pathway (11). A resting T-cell might withdraw CTLA4 from its surface via endocytosis with the clathrin adaptor protein AP-2 when not interacting with an APC. Members of the Src family of kinases (including lck, fyn, and rlk) prevent this by phosphorylating CTLA4 intracellularly to prevent this association (13).

At the immune synapse, ligation of TCR causes formation of microclusters of kinases and adapter proteins needed to transmit signals for activation and cell survival, these include ZAP-70, SLP-76, and Gads (Grb2-related adapter downstream of SHC). Coligation of TCR and CTLA4 blocks the formation of these microclusters and inhibits the translocation of calcium needed to cause proliferation of the T-cell. Similarly, CTLA4 binding blocks the formation of lipid rafts, also called glycolipid-enriched microdomains. Produced from TCR and CD28 coligation, these rafts are formed at the membrane and carry signaling proteins. Blockade of both the rafts and microclusters are hypothesized to affect downstream transcription factors such as NF-kB, NFAT, and AP1, inhibiting cell cycle and proliferation (11).

A final component of CTLA4 binding involves its interruption of the TCR stop signal. Binding of the TCR with the antigen of an APC prompts the transmission of a “stop signal” in the T-cell, which reduces cell motility and increases adhesion. This prolongs the interaction between the T-cell and APC, allowing for the adequate time needed for antigen recognition and T-cell activation. CTLA4 is thought to override this signal produced by coligation of TCR and CD3 (another coreceptor involved in activation) and thus prevents proliferation and activation (14).

Although many studies focus on the role of the T_eff_ cell in studying CTLA4, the function of T_reg_ cells is just as important. T_reg_ cells constitutively express CTLA4 and respond to activation with immunosuppressive effects; knockout of CTLA4 on these cells has shown to significantly increase antitumor effects. The cell signaling mechanism produced by CTLA4 is not totally understood in either T-cell variety and is likely different between the two, revealing the complexity behind the workings of an immune checkpoint inhibitor.

##### Ipilimumab (YERVOY^®^, Bristol-Myers Squibb)

Ipilimumab (YERVOY^®^) is a CTLA4 inhibitor that prevents CTLA4 interaction with CD80 and CD86. In 2011, it became the first immune checkpoint inhibitor to be approved by the FDA. Its fast tracked approval came following phase III trial results showing its efficacy against advanced melanoma. Prior to the approval of ipilimumab, no existing drug therapy had shown improved overall survival (OS) for advanced melanoma. The first phase III trial treated patients with ipilimumab 3 mg/kg, based on safety and efficacy studies in phase II trials, and was compared to glycoprotein 100 (gp100) a melanoma peptide vaccine. The result was a near doubling in 1 and 2 years OS (15). Phase II trials also showed promising results when ipilimumab was used to treat a subset of melanoma patients with brain metastases. These trials showed an equal odd ratio when treating both visceral lesions or within the brain, suggesting ipilimumab is effective at crossing the blood brain barrier. When combined in these trials with fotemustine, a nitrosourea alkylating agent, treatment achieved a median OS of 13.4 months and 1 year survival rate of 54.2% (16). Prior to the approval of ipilimumab, patients with advanced melanoma had a 5-year survival rate of approximately 10%. By 2013, several phase II trials had reached the 5-year mark and showed survival rates as high as 28.4% in those previously treated, and as high as 49.5% in treatment naïve patients (15). The most common side effects are immune related and occurred in about 60% of patients in phase II and III studies. These were mostly low grade and the majority were skin conditions such as pruritus and rash, or GI conditions such as diarrhea and colitis. Studies are also underway investigating the efficacy of ipilimumab in combination to treat NSCLC, small-cell lung cancer, castration resistant prostate cancer, metastatic bladder cancer, and other cancers.

#### Programmed Cell Death Protein 1 (PD-1)/PD-L1 Inhibitors

Programmed cell death protein 1 is another immune checkpoint that saw success as a drug target following CTLA4. Similar to CTLA4, PD-1 binding causes inhibition in T_eff_ cells and enhancement in T_reg_ cells. Unlike CTLA4, the PD-1 receptor binds B cells and myeloid cells in addition to T-cells. Its ligands, PD-L1 and PD-L2 are also widely expressed among leukocytes, myeloid-derived suppressor cells (MDSCs) and cancer cells themselves. Binding is thought to initiate a signaling cascade involving SHP2, which inhibits downstream Ras and PI3K/Akt survival pathways (Figure 1). The focus of this checkpoint is usually on the interaction with PD-L1, which has higher binding affinity. PD-1 is thought to regulate immune cells after they have been activated, while CTLA4 regulates activation itself. PD-1 is not found on resting T-cells, but upregulation occurs rapidly upon activation via AP-1 binding to the associated gene promoter (17). The mechanisms of PD-1 action are similar to CTLA4 and include inhibition of pro-survival and proliferation pathways, and the upregulation of the BATF (Basic leucine zipper transcription factor ATF-like) protein, which normally occurs in response to T-cell exhaustion as a method of avoiding autoimmunity (18). The interruption of microclusters is also shown to occur. PD-1 binding induces a conformational change that allows for intracellular phosphorylation via Lck. This recruits phosphatases SHP1 or SHP2, which then inhibit the aforementioned microcluster signaling molecules like ZAP-70, CD3, PI3K, and PKCθ (19). The combined result is reduced proliferation and cytokine secretion. PD-1 activation is also found to inhibit TCR activation of survival pathways mediated by PI3K/Akt/Ras-MEK/ERK as well as S-phase kinase-associated protein 2 (SKP2). Inhibition of SKP2 results in inhibition of CDK2, which then inhibits downstream effectors that in turn further inhibit SKP2. The result is a negative feedback loop on the cell cycle leading to apoptosis (20).

##### Pembrolizumab (KEYTRUDA^®^, Merck & Co)

Pembrolizumab (KEYTRUDA^®^) is a fully humanized antibody targeted against the PD-1 receptor. FDA approval was fast tracked after it was deemed a breakthrough therapy based on results of a phase IB study for treatment of metastatic unresectable melanoma. The study included two treatment arms dosed at 2 and 10 mg/kg. Participants (*n* = 173) were those who had not responded to treatment with ipilimumab or BRAF inhibitors. The primary endpoint was the overall response rate (ORR), which was estimated at 24% in both treatment arms. The estimated 1-year survival rate was 58% in the 2 mg/kg treatment group and 63% in the 10 mg/kg treatment group (21). Among the 82% of patients who were reported to develop an adverse reaction during therapy, only 12% were of grade 3 or 4 in severity. The most common reactions were fatigue, pruritus, and rash, which were well managed with corticosteroid treatments without the need for discontinuing therapy. Other rare but more severe reactions also occurred such as: pneumonitis, occurring in 3% of participants at a median time of 5 months into therapy, and thyroid disorders in about 9.5% of participants (21). Pembrolizumab is also being studied in many other cancers including NSCLC, head and neck squamous cell cancer, gastric cancer, triple negative breast cancer, and colorectal cancer (CRC).

##### Nivolumab (OPDIVO^®^, Bristol-Myers Squibb)

Nivolumab (OPDIVO^®^) is another fully humanized antibody directed against the PD-1 receptor. Similar to pembrolizumab, it was granted accelerated approval by the FDA in 2014 for the treatment of metastatic or unresectable melanoma unresponsive to ipilimumab or BRAF inhibitors. In 2015, it was also approved for metastatic squamous NSCLC unresponsive to platinum-based chemotherapy. The benefits for which nivolumab was approved are all summarized in a second phase III study (22) conducted on 418 patients who were naïve to any anticancer therapy and without regard to BRAF mutation status. In this study, patients were randomized 1:1 to two treatment arms which compared nivolumab 3 mg/kg every 2 weeks to dacarbazine 1,000 mg/m^2^ every 3 weeks. The nivolumab treatment group showed an ORR of 40% compared to 13.9% with dacarbazine. Nivolumab also produced a median progression-free survival (PFS) of 5.1 months and 1 year OS of 72.9%, compared to 2.2 months and 42.1% for dacarbazine, respectively (22). As seen in the first phase III study, grade 3 or 4 adverse events were also less frequent with nivolumab treatment, 11.7% compared to 17.6% for dacarbazine. The most common side effects were fatigue, rash, diarrhea, and musculoskeletal side effects (22).

Pembrolizumab and nivolumab are often options employed in patients unresponsive to ipilimumab. Some studies have shown consistently better outcomes in advanced melanoma compared to ipilimumab, but most importantly, studies combining the two seem to confirm the possibility of synergism, despite their very similar underlying molecular pharmacology. Since the field of I-O is new and expanding, combination therapies have yet to be significantly explored, but early investigations such as these show great promise.

##### Atezolizumab and Future PD-L1 Inhibitors

Atezolizumab (Tecentriq, Genentech) was the first FDA approved drug among the class of PD-L1 inhibitors implicated for the targeted treatment of bladder cancer. Another drug in development, BMS-936559/MDX1105 (Bristol-Meyers Squibb), which also binds to CTLA4 and CD28 is shown to be effective in a Phase I trial in patients with advanced cancers including NSCLC, kidney cancer, and melanoma (23). Some other drugs in this class including MEDI4736 (MedImmune), MPDL3280A (Roche), MSB0010718C (Merck) have shown promising results in Phase I trials of NSCLC patients.

### Small Molecules

Still lacking in the field of I-O is an emphasis on small molecule drugs, which are used in the treatment of many cancers. Small-molecule drugs are desirable due a few distinct advantages: many are well studied and highly characterized in terms of PK/PD (24), they can access intracellular targets that large protein counterparts cannot, and can more easily cross physiological barriers. Moreover, they are attractive to patients because the therapies are more often orally bioavailable and avoid uncomfortable routes of administration, and they are often significantly less expensive than other options in the field.

#### Enzyme Inhibitors

The breakdown of certain amino acids has been implicated in the regulation of immune responses to cancer. Metabolites of tryptophan by enzymes like indoleamine-pyrrole 2,3-dioxygenase (IDO) have been shown to induce the production of T_reg_ cells that suppress the immune system. Likewise, the breakdown of arginine results in poor display of TCRs on the cell surface. There is evidence that tumor cells overexpress the enzymes responsible for these products and as a result, inhibitors have been developed for clinical trials. Enzymes targeting arginine include inducible nitric oxide synthases and ARG1 and both are highly expressed in MDSC, which are immunosuppressive cells common in the tumor microenvironment. Phosphodiesterase type 5 (PDE5) inhibitors, such as tadalafil (Eli Lilly), inhibit the degradation of cGMP in MDSCs, resulting in reduced expression of the above enzymes downstream (24). Tadalafil is an already heavily marketed PDE5 inhibitor used to treat erectile dysfunction and a phase II trial published in 2015 explored the expansion of its use in treating head and neck squamous cell carcinoma. Tadalafil showed a significant increase in T-cell expansion (2.4- vs 1.1-fold) and a decrease in peripheral MDSCs (0.81- vs 1.26-fold change) when compared to placebo (25).

#### COX2 and PGE2 Receptors

While many I-O therapies induce acute inflammation for tumor cell killing, chronic inflammation has been repeatedly linked to tumor development and growth in a variety of cancers. The COX enzymes, promoters of chronic inflammation, have been implicated in cancers such as breast, colorectal, and lung (26–28). COX levels are found to be elevated in the hypoxic environment developed by a growing tumor, and such elevation has been shown to correlate with lower survival rates (29). COX2 metabolites such as PGE2 also contribute to immunosuppression through their activation of MDSCs and T_reg_ cells. COX-2 inhibitors are already on the market with many being particularly inexpensive, so expanding their use into cancer therapy would make them a valuable tool. A study performed with Celecoxib, a selective COX2 inhibitor approved for the treatment of pain and certain inflammatory diseases, was used in an *in vivo* mouse model of spontaneous breast cancer where it showed a significant survival improvement compared to placebo (30). Small molecules designed to inhibit PGE2 or its downstream effectors such as adenylyl cyclase and cAMP are now under investigation.

#### Toll-Like Receptors (TLRs)

Toll-like receptors are expressed on the surface of APCs, leukocytes, and the cells of various non-immune-related tissues where they bind bacterial, viral, and fungal antigens and produce a cytokine-mediated immune response via dendritic cells and NK cells. This inflammatory response has been manipulated to treat cancer through the development of small molecule TLR agonists. One such pharmaceutical is Imiquimod, a TLR7 and TLR8 agonist that is FDA approved for the treatment of basal cell carcinoma (BCC). The first study in 1999 of topical 5% Imiquimod in patients with BCC demonstrated the promise of small molecule monotherapy. In a phase II study, of the 35 patients enrolled, over 60% showed a complete clearance of the carcinoma (31). Other compounds under investigation also include analogs of lipopolysaccharides (LPS), a natural TLR4 agonist found in gram negative bacteria. Evidence also exists that TLR activation can re-activate APCs in the tumor microenvironment and aid effector T-cell function (24). Recent clinical studies have explored specific TLR agonists in combination with other targeted cancer therapies (32). One such Phase Ib clinical study (NCT00633529) was conducted in NSCLC patients using increasing doses of the TLR9 agonist IMO-2055 (Idrea Pharmaceuticals & Merck) in combination with erlotinib and bevacizumab; reporting that the drug was well tolerated and progressed to the next phase of clinical trials (33). Similarly, another TLR9 agonist, MGN1703 was evaluated in a Phase II study (NCT01208194) in metastatic CRC patients on first line chemotherapy and indicated an improved PFS compared to placebo control patients (34).

#### Chemokines

Chemokine signaling in the tumor microenvironment tends to be associated with immunosuppression. Although efforts have been made in developing mAbs targeting chemokines such as CCL2 (CNTO888) (35), small molecules designed to antagonize chemokine receptors offer a viable approach as well. The CXCR family of receptors, known primarily as the target for HIV viral attachment and entry, is also found overexpressed in a variety of cancers and contributes to elevation of regulatory T-cells. Plerixafor is a C-X-C chemokine receptor type 4 (CXCR4) antagonist, given orphan drug status by the FDA in 2008 for the treatment of lymphoma and myeloma through mobilization of hematopoietic stem cells. However, use in non-Hodgkin lymphoma has also shown it to increase levels of neutrophils, lymphocytes, and monocytes (36). CCR5, also a receptor involved in HIV infection, has been shown to signal infiltration of immunosuppressive cells such as MDSCs when stimulated in the tumor microenvironment. Maraviroc is a CCR5 antagonist approved for the treatment of HIV, but is now being explored in phase I trials for the treatment of CRC (24).

#### Signal Transduction

Intracellular signaling pathways have already been successfully exploited for cancer therapeutics, and their effects in the context of I-O are now being more clearly defined. Using signal transduction cascades as a drug target is attractive due to the variety of pathways and messengers that could be viable targets. One such strategy targets the colony stimulating factor (CSF) pathway. CSF binding at its receptor causes tyrosine kinase-mediated autophosphorylation that leads to proliferation of tumor-associated macrophages (TAMs), which inhibit immune responses and assist tumor invasion. Early clinical evidence from CSF1 inhibitors has shown evidence of slowing tumor growth. Gleevec (Imatinib) is a famous agent in this drug class, approved in 2001 for chronic myeloid leukemia and which marked a huge step forward in cancer therapeutics. While developed as a tyrosine kinase inhibitor, it also inhibits the KIT oncogene. KIT activation has been connected to expression of IDO, mentioned earlier in regards to its immunosuppressive function. Consequently, treatment of patients with gastrointestinal stromal tumors using Imatinib has resulted in an increase in PFS with enhanced production of IFNy and NK cells, which can be attributed to this additional mechanism of action (24). These off-target immunotherapeutic effects have been seen in other approved targeted therapies, including MEK and BRAF inhibitors. The effects are similar, with decreased MDSC counts and increased CD8+ T-cells found at the site of the tumor.

## Active Therapy of Biological I-O Treatments

Active therapies direct the body’s immune cells to attack TAAs, stimulating them to recognize, and destroy the cancer.

### Cell Therapy or Adoptive Cell Transfer (ACT)

**Figure 2:**
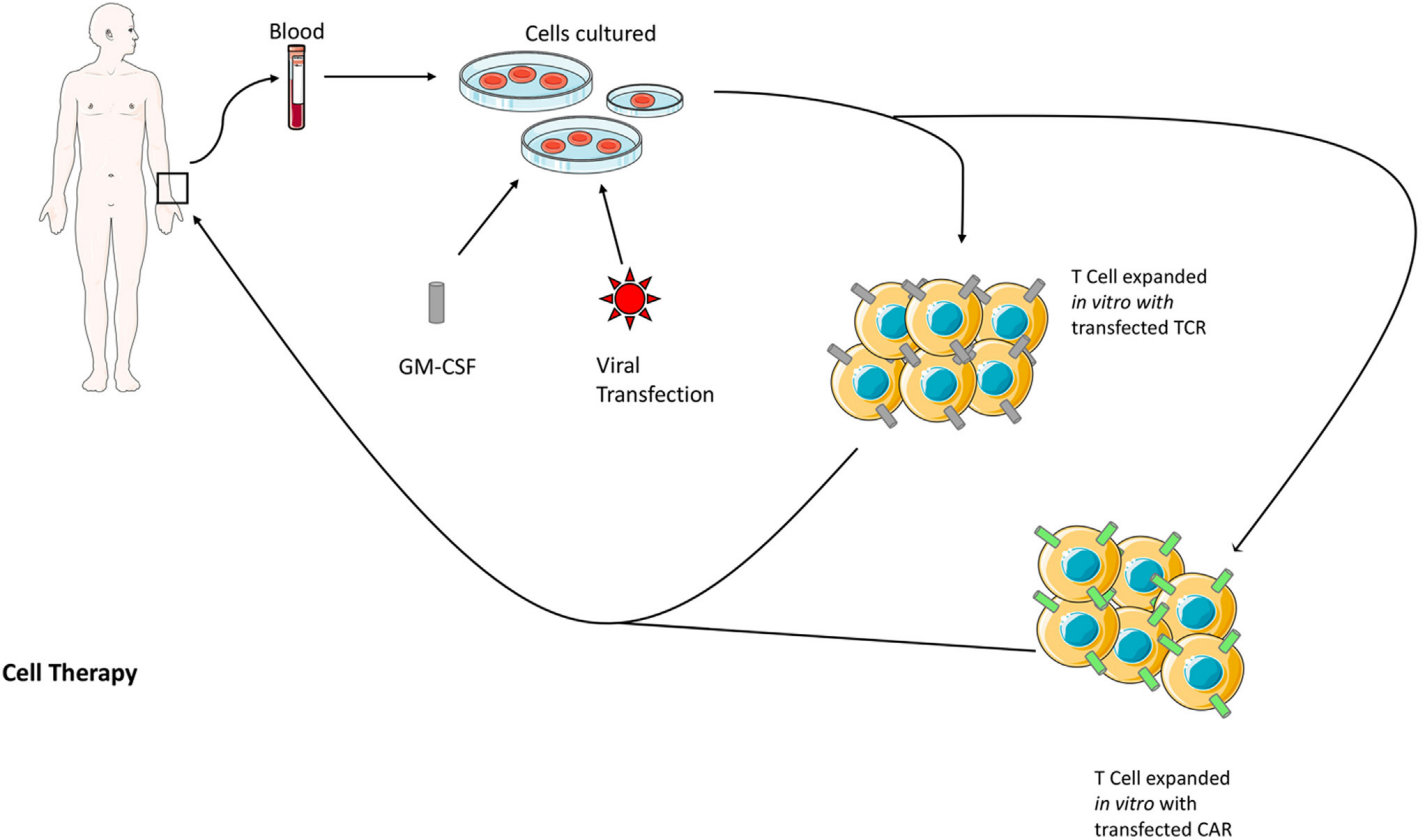
**The two methods of genetically modified ACT are shown above**. Both methods begin with the isolation of TILs from the patient’s blood, which are then expanded *in vitro* with the growth factor GM-CSF. At this stage, the cells are also transfected with a virus encoding either the genetically modified TCR or CAR. The modified TILs are then reintroduced to the patient. Abbreviations: ACT, adoptive cell transfer; CAR, chimeric antigen receptor; GM-CSF, granulocyte–macrophage colony-stimulating factor; TCR, T-cell receptor; TILs, tumor-infiltrating lymphocytes. Some elements adapted from Servier Medical Art (https://creativecommons.org/licenses/by/3.0/legalcode).

The practice of ACT was born of other key findings in the recent strides in I-O. The discovery of immune-surveillance and the utility of IL-2 therapy brought forth the hypothesis that T lymphocytes could be extracted from a patient, expanded *in vitro*, and re-administered as a cancer therapy (Figure 2). This was achieved in humans in 1988, where researchers used an expanded line of tumor-infiltrating lymphocytes (TILs) to produce regression in patients with metastatic melanoma (37). ACT works by resecting a tumor specimen and digesting it into a single-cell suspension. The suspension is then separated into individual cultures which are treated with high dose IL-2 that expands TILs and results in the destruction of remaining tumor tissue within 2–3 weeks. The resulting pure cultures of lymphocytes are then tested for tumor recognition (37). A colorimetric assay is used to measure the levels of interferon gamma released by the culture in response to tumor antigen which will correlate with their activity *in vivo*. Potent cultures move on to a final process called rapid expansion, where they are treated in a medium again containing high dose IL-2 that results in a >1,000-fold expansion; the resulting lymphocytes, which can number in the billions, are then administered to the patient.

Although promising, initial studies in ACT were not without drawbacks; responses were short lived and the transferred cells did not persist *in vivo* for very long. Immune suppressor cells like T_reg_ and MDSCs attenuated the activity of transferred cells by outcompeting them for the necessary cytokines. The solution was lymphodepleting pretreatment in the form of chemotherapy (typically a combination of cyclophosphamide and fludarabine) or radiation therapy that eliminated the suppressor cells allowing the TILs to persist (38). Lymphodepletion proved to greatly enhance the outcomes of ACT, transferred cells persisted in the body for months and in the treatment of metastatic melanoma some patients showed complete regression and were possibly cured (37). The other drawback to conventional ACT was that it depended upon extracting TILs from the tumor environment because those had been naturally developed to recognize TAAs, this meant surgical resection. Some tumors lack a high degree of TILs at all, a reason why the initial studies into ACT were specifically performed on metastatic melanoma because of the high rate of mutagenicity associated with this cancer and therefore its propensity to develop TAAs and attract TILs. In response to this, two new methods of ACT surfaced: TCR, and chimeric antigen receptor (CAR) approaches that were both evaluated in several clinical trials (39).

In TCR therapy, normal circulating T-cells are isolated from the patient’s blood and genetically modified via transfection with a retrovirus vector or transposon to express TCRs against a tumor antigen (38). Specific TCRs are gathered from human patients or mice immunized against the TAA of interest. The technology saw its first clinical trials in 2006 and has allowed for the expansion of cell therapy beyond the realm of metastatic melanoma; clinical responses have been produced targeting antigens such as CD19 in B-cell lymphoma and carcinoembryonic antigen (CEA) in CRC (38).

The limitation of TCR therapy is that the recombinant TCRs still rely upon MHC recognition to achieve cytotoxicity. This is a problem considering downregulation of MHC I is a major method of tumor immune evasion and that 40–90% of human tumors derived from various MHC class I + tissues were reported to be MHC I deficient (40). Chimeric antigen receptor technology was developed and introduced in 2010 to circumvent this issue. This method again utilizes transfection through a virus vector but introduces an antibody variable region to the T-cell, which will be expressed on the membrane and linked to intracellular signaling domains. The first CARs linked to CD3-zeta and the method was later improved to involve costimulatory receptors such as CD28, OX40, and more.

Melanoma quickly emerged as the perfect target for ACT therapy due to its aforementioned high mutation rate resulting in many tumor-specific antigens. The success in using ACT therapy and other I-O methods in treating melanoma brought forth the hypothesis that TILs can and do target individual mutations specific to the tumor. ACT therapy has now been expanded further to manipulate this. Both healthy and cancerous cells are extracted from a patient and sequenced to identify tumor-specific mutations; mini-genes corresponding to the mutated peptide sequence can then be synthesized which are electroporated into a population of APCs (37). The APCs display the tumor antigens on their MHC and patient’s extracted TILs are cocultured for recognition, with the remaining steps proceeding as explained previously. Although this method further limits ACT therapy to high mutagenic cancers, it promises stronger responses and tumor regressions for those who are applicable.

Clinical trials using ACT therapy have resulted in patient responses that show much promise for the future. Metastatic melanoma has historically been the prime target where treatments are few and often ineffective. Results of a trial published in 2011 using ACT therapy supplemented with lymphodepletion in patients with metastatic melanoma showed an objective response rate as high as 72%. Of the 93 patients within the three trials studied, 19 (22%) achieved complete regression with all but one of those patients having ongoing regression after three years. Of the 19 patients, 70% had progressed to ACT therapy after failing to respond to IL-2 therapy, chemotherapy, or both (41). Results of a trial published in 2014 showed equal promise for CAR. About 30 patients with acute lymphoblastic leukemia were treated with CD19 CAR therapy, and 27 (90%) showed complete remission, with 67% of those sustaining at 6 months (42). A clinical trial testing TCR therapy targeting the NY-ESO-1 antigen on six patients with metastatic melanoma or refractory metastatic synovial cell sarcoma was published in 2011 and is to date the first trial using ACT in a non-melanoma solid tumor. Objective responses were seen in four of the patients, one of which continued a partial response for 18 months (43). Although small, this trial was the first evidence that ACT can be effectively expanded beyond treatment of only melanoma, and demonstrates the growth still to come in this field.

The biggest challenge to the future of ACT therapy remains toxicity. Tumor-specific antigen targets will help avoid such toxicity, which could result in life-threatening immune reactions. The early melanoma antigen targets gp100 and MART-1 are found on both melanoma cells and healthy melanocytes, and thus early ACT trials were met with severe eye and skin toxicity where melanocytes are found (37). Even with novel antigen targets safety is not guaranteed; A clinical trial published in 2013 used TCR therapy to target the MAGE-A3 antigen unique to cancer cells. Two of the nine patients in the study died due to cross reactivity when TILs recognized the related MAGE-A12 protein found in the brain (44). In cases such as these, the lymphodepletion meant to enhance the TIL activity instead serves to further the toxic effects. Regardless of the target, patients are continually at risk of developing cytokine release syndrome (CRS). The infusion of billions of lymphocytes simultaneously into the patient results in their overwhelming release of cytokines such as INFγ and interleukins that can cause fever, fatigue, cardiac, liver, and renal toxicity, and eventually organ failure. This is the most common side effect of CAR therapy and in the 2014 CAR trial mentioned above, occurred in every patient in the study to some degree, with 27% experiencing severe cases that required intensive care (42). Genetic engineering remains the solution to these setbacks. Advancement of CAR and TCR using TILs sensitized to tumor-specific antigens will be the future, and at present gives enthusiasm for ACT to become both safe and effective.

### Cancer Vaccines

The discovery that lymphocytes could selectively target and attack cancer cells based on TAAs opened the possibility of developing therapeutic vaccines to treat cancer. In the broad context of I-O cancer vaccination has seen slower growth and more challenges compared to other approaches. Underwhelming clinical trial results have hampered success in some areas but numerous clinical trials are underway (see Table 1) that could fare much better. Vaccines are classified based upon how they present the antigen and there are several methods for this.

**Table 1:** **Summary of promising I-O therapies in development**.

Summary of completed phase I trials in the field of immuno-oncology				
Target/type	No. of completed phase I trials^a^	Example		Reference
		Agent/drug	Condition	
**PASSIVE THERAPY**				
**Monoclonal antibodies**				
Cytotoxic T-lymphocyte associated protein 4 (CTLA4)	22	MDX-CTLA4 antibody (45)	Melanoma	NCT00028431
Programmed cell death protein 1/PD-L1	8	Nivolumab	Neoplasms	NCT01629758
PD-L1		Atezolizumab	Bladder cancer	Approved
**Small molecules**				
Indoleamine-pyrrole 2,3-dioxygenase	7	INCB024360 (46)	Advanced malignancies	NCT01195311
Toll-like receptors (TLR)	34	TLR8 Agonist VTX-2337 (47)	Ovarian cancer	NCT01294293
CXCR	26	Anti-C-X-C chemokine receptor type 4 (BMS-936564) (48)	Multiple myeloma	NCT01359657
BRAF	26	ARQ 736	Solid tumor	NCT01225536
**ACTIVE THERAPY**				
**Cancer vaccine**				
PSA/Adenovirus	2	CV787 (CG7870) (49)	Prostate cancer	NCT00116155
gp100, MAGE-3	3	gp100 antigen/recombinant MAGE-3.1 antigen	Melanoma (skin)	NCT00003792
HER-2^b^	22	E75 + granulocyte–macrophage colony-stimulating factor (GM-CSF) vaccine (50, 51)	Breast cancer	NCT00854789
**Cell therapy**				
NY-ESO-1	28	NY-ESO-1 protein/CpG	Prostate cancer	NCT00292045
CD123	1	CSL362 (52)	Leukemia, myeloid, acute	NCT01632852
CD19	25	SAR3419	Lymphoma; non-Hodgkin	NCT00796731
**Oncolytic viruses**				
Parvovirus	1	H-1PV (53)	Glioblastoma multiforme	NCT01301430
Adenovirus^c^	49	Adenoviral vector encoding rat HER-2/neu	Metastatic/recurrent breast cancer	NCT00307229
Herpes simplex viruses (HSV)-1^d^	18	Recombinant hGM-CSF HSV Injection	Melanoma; liver cancer; pancreatic cancer; lung cancer	NCT01935453
**Bispecific T-cell engagers**				
Prostate-specific membrane antigen (PSMA)	10	PSMA antibody drug conjugate (54)	Prostate cancer	NCT01414283
Carcinoembryonic antigen (CEA)	38	Anti-CEA second generation designer T-cells (55)	Liver metastases	NCT01373047

*^a^Information from http://clinicaltrials.gov using advanced search (search terms, recruitment “Completed,” Additional Criteria “Phase 1”)*.

^b^Search terms used “HER-2 cancer vaccine.”

^c^Search terms used “Adenovirus Cancer.”

^d^Search terms used “HSV-1 Cancer.”

#### Dendritic Cell Vaccines

**Figure 3:**
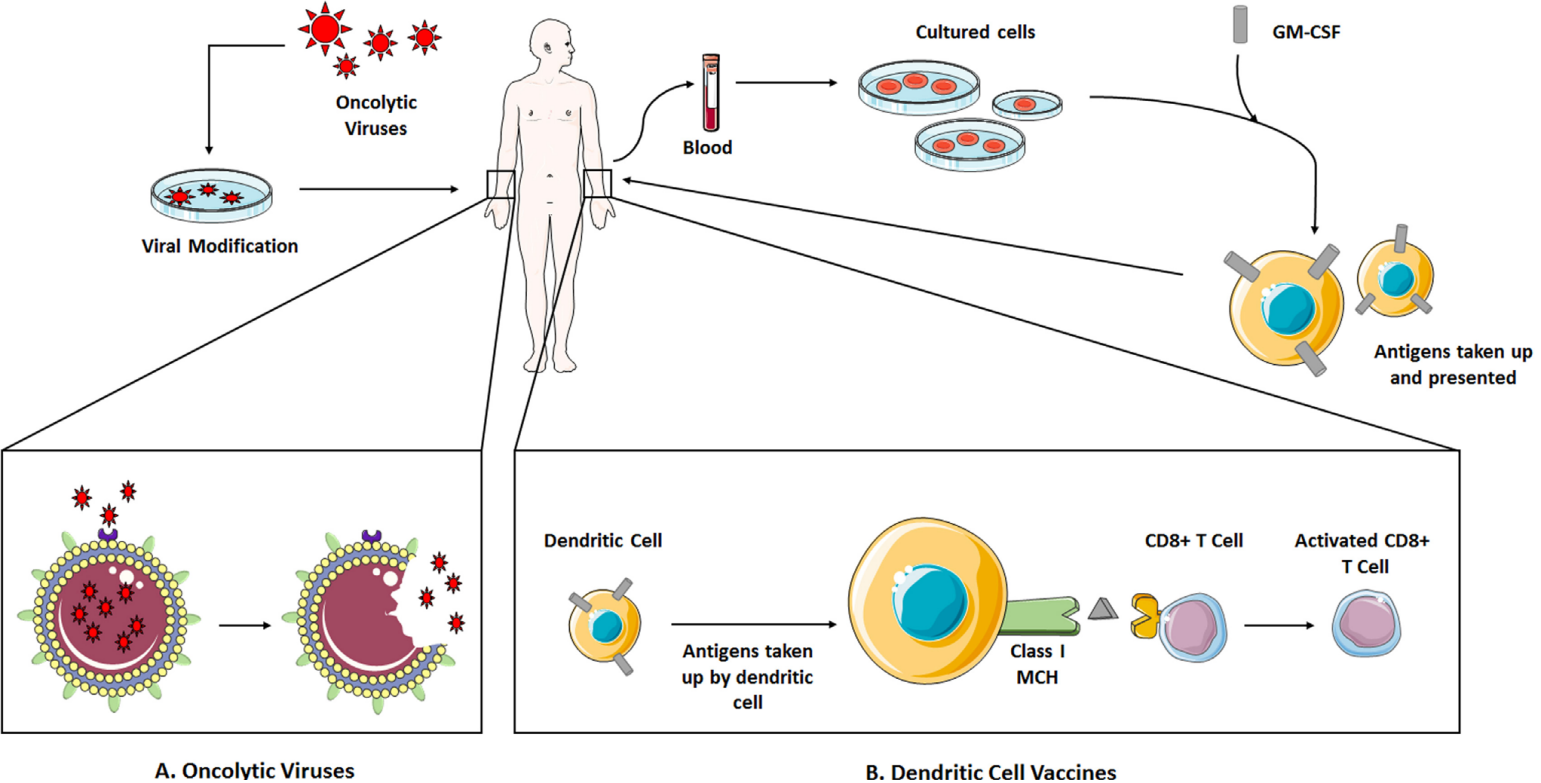
**(A)** Naturally occurring viruses undergo genetic modification to reduce pathogenicity toward healthy cells while increasing selectivity for tumor cells, becoming OVs. They are introduced to the patient where they infect and kill tumor cells, and spread. **(B)** The MOA of a dendritic cell vaccine, an example of a cancer vaccine. Dendritic cells are isolated from the patient’s blood and incubated with the selected TAA and GM-CSF for expansion. They are reintroduced to the patient where they present the TAA to CD8 + T-cells in the context of MHC I, activating these T-cells for tumor destruction. Abbreviations: GM-CSF, granulocyte–macrophage colony-stimulating factor; OVs, oncolytic viruses; TAAs, tumor-associated antigens. Some elements adapted from Servier Medical Art (https://creativecommons.org/licenses/by/3.0/legalcode).

Dendritic cell vaccines can be considered to be the most successful of approaches. Dendritic cells are APCs in the body that engulf foreign molecules and present their antigens in the context of MHC II for recognition by T lymphocytes to start an adaptive immune response. For production of a vaccine, these cells are extracted from a patient and incubated with the selected antigen and adjuvants. Granulocyte-macrophage colony stimulating factor (GM-CSF) is then introduced to expand the cell population, which is then administered back to the patient (Figure 3).

Sipuleucel-T (PROVENGE^®^), manufactured by Dendreon Corporation, is an FDA approved dendritic cell vaccine for treatment of prostate cancer, and the only approved vaccine to date. Its approval came in 2010 following the results of a phase III clinical trial where it demonstrated a 4.1-month improvement in OS compared to placebo (interestingly, this was without any difference in time to progression) (56). Sipuleucel-T targets prostatic acid phosphatase, found in almost all prostate cancer cells, which is presented to dendritic cells as a fusion protein combined with GM-CSF. The future of dendritic cell vaccines is likely to involve genetic modification. T-cell activation is highly dependent upon costimulatory proteins expressed on the APC surface, including CD40, CD70, and OX40 (56). Genetically upregulating these *in vitro* during the incubation process, in addition to downregulation of inhibitory proteins, can enhance the efficacy of future vaccines. As this article will explore, the future of dendritic cell vaccines and all cancer vaccine subtypes is likely to depend heavily on similar genetic modifications.

#### Whole-Cell Tumor Vaccines

The use of whole tumor cells offers another vaccine approach. Cancer cells are resected from the tumor and then attenuated. This can be through several methods including UV irradiation, freeze thawing, or heat shock (57). Each of the processes leads to the release of proteins by the tumor cell (either at the cell surface or exogenously) that will be later recognized by the body’s APCs. Attenuation also stops the cell from releasing inhibitory cytokines that would dampen the immune response by T lymphocytes. The attenuated tumor cell is combined with adjuvants and then reintroduced for recognition by the immune system much like a typical vaccine. The advantage to this method is that the whole intact cell allows for the complete array of TAAs possible to be presented to the immune system. Autologous tumor cells provide a personalized vaccine, which is significant considering it has been suggested that up to 95% of the mutations within an individual tumor are patient specific (58). The drawback is that the procedure requires a solid tumor that is surgically reachable. The tumor cell need not be autologous, allogeneic tumor cell vaccines present the possibility of an even wider array of antigen presentation and are easier to produce. The use of various different adjuvants have been applied to this method, LPS can be included to stimulate the immune response, or tumor cells can be genetically engineered to express cytokines that promote proliferation of immune cells such as GM-CSF, IL-2, and Ftl3 (56). A popular adjuvant also used in whole tumor vaccines is bacille Calmette–Guérin (BCG). Isolated from *Mycobacterium bovis*, BCG has been used decades worldwide as a vaccine against tuberculosis (59). By the 1970 s, it was discovered that administration of BCG alone into the tumor environment was an effective treatment for bladder cancer; the interaction between the pathogen-associated molecular patterns and lymphocyte pattern recognition receptors prime an immune response that can then act upon the tumor. Several whole-cell tumor vaccines have entered clinical trials but, representative of the cancer vaccine field as a whole, results were often not as promising as hoped. Canvaxin was an allogeneic tumor cell vaccine that entered phase III clinical trials in 2004 for treatment of melanoma, after failing to show a survival benefit the trials were ended early and the vaccine was abandoned (56). The prostate GVAX vaccine, also using allogeneic tumor cells, has seen better results and significantly improved OS in phase I and II trials (56). These trials also reported a lack of autoimmune toxicities, implying that the safety of vaccines could make them an attractive option in the future.

#### DNA/RNA-Based Vaccines

DNA/RNA-based vaccines are also being tested in clinical trials. DNA vaccines contain the requisite TAAs encoded onto a bacterial plasmid which is injected into the patient. The plasmid is up-taken by local cells, including APCs which naturally move to the site of the injection, once up-taken, the APCs can then directly produce their own antigen. While conceptually promising, DNA vaccines have yet to show benefit when tested in clinical trials. Alternatively, the genetic info can be in the form of RNA, which undergoes rapid enzymatic degradation and therefore avoids any possible toxicity. RNA vaccines are also currently in clinical trials.

#### Protein/Peptide Vaccines

Similar to classic vaccines, therapeutic cancer vaccines are also made using just protein/peptide antigens. The TAAs used can vary and one vaccine can contain multiple antigens. Applications have been found for breast, testicular, and prostate cancer, as well as melanoma. While easier to manufacture than whole-cell vaccines, peptide vaccines present a much smaller array of antigens to the host immune system. However, upon employing genetic modification, the peptide sequence of epitopes in these vaccines can be modified to enhance antigenicity and therefore promote binding to T lymphocytes. The gp100 vaccine for the treatment of metastatic melanoma is an example of such and one of the more researched peptide vaccines to date. The protein gp100 is a transmembrane glycoprotein and features an amino acid substitution in the peptide sequence for enhanced lymphocyte binding. Gp100 has reached phase III clinical trials but results have been mixed, those studies showing therapeutic benefit have not been reliably produced, and in most trials it is used in combination with another I-O therapy such as IL-2 therapy or immune checkpoint inhibitors to provide a patient benefit (56). Adjuvants for these vaccines are similar to those used for the whole-cell vaccines such as BCG and LPS; bacterial or viral antigens seem to be the most effective at eliciting an immune response. Aluminum salts are also a very common adjuvant for these vaccines and have been used for many years, it is theorized that aluminum can activate APCs directly and/or form conjugates with the administered TAAs that increase their uptake (59).

#### Viral-Based Vaccines

Viruses are naturally potent at stimulating an immune response, making them ideal not just as adjuvants but as the complete vector for the vaccine. TAAs are encoded into an attenuated virus, which will then transduce host cells and lead to expression of the antigen; among the transgenes, IL-2 and GM-CSF can also be encoded as in previously mentioned vaccines to increase lymphocyte proliferation.

The high replication rate of viruses compared to living cells provides an advantage as a vector. Members of the poxviridae family (poxviruses) are the most popular because they can accommodate large amounts of genetic info, are stable, and replicate with accuracy (56). Attenuation occurs through deletion of pathogenic genes, or in the case of the MVA vaccine (modified vaccinia virus), numerous *in vivo* serial passages resulting in a weakened viral genome (56); either method produces a strain that can infect cells and cause protein expression but cannot damage the host. Modified vaccinia ankara vaccine has the advantage of being safe for use in immunocompromised patients. Gene expression using poxviruses can occur for about 1–3 weeks and occurs without the risk of insertional mutagenesis. Poxvirus vectors are used as the priming vaccine but due to the adaptive host immune response, will not provide further benefit with repeat administration, therefore, boosters are given using avipoxvirus vectors, which do not elicit the production of neutralizing antibodies in mammals and allow for the continuation of therapy. Viral vector technology has been extended even further with TRICOM, a formulation of transgenes for the treatment of prostate cancer that combines prostate-specific antigen and three different T-cell costimulatory molecules to enhance the immune response. The prime and boost method, in combination with TRICOM, was used together in a therapy termed PROSTVAC, and underwent a 43-center, 125-patient randomized placebo controlled trial. Although failing to achieve clinical endpoints, the treatment arm did see a significant improvement in OS (60). TG4010 is an MVA type vaccine with transgenes expressing the antigen MUC-1 and cytokine IL-2. A phase II trial published in 2015 combined TG4010 and cytokine therapy for treatment of renal carcinoma. Results showed an improved OS but no clinical response (61).

### Oncolytic Viruses (OVs)

The use of viruses in I-O has been taken one step further by using them not as the vector for therapy but as the drug therapy agent itself. These OVs are engineered to selectively infect and kill tumor cells without the pathogenicity to healthy cells seen in a normal virus. (Figure 3) The idea for using viruses as a cancer treatment has existed for a few decades but only now has become feasible due to advances in genetic technology.

Production of an OV focuses on safety, adherence to quality and purity, and achievement of a high enough titer. Herpes simplex viruses (HSV), adenoviruses, measles viruses, reoviruses, vaccinia viruses, and more have all been utilized as treatments. Like the aforementioned viral vectors, OVs undergo deletion of genes to reduce their pathogenicity to healthy cells while preserving their ability to replicate *in vivo*. An example is the deletion of the viral gene encoding thymidine kinase in HSV. This enzyme is critical for DNA synthesis and is expressed by the virus but also in proliferating human cells. Deletion of the gene in the virus makes it dependent on the host enzyme to replicate and therefore causes it to preferentially target rapidly proliferating tumor cells (62). Selectivity is further enhanced by modifying viral attachment proteins to target TCRs. Modifying the viral genome to make a virus able to replicate only in rapidly proliferating cells is the main method of engineering these viruses to selectively target the tumor. Once the genetic modification of the virus is complete, manufacture is a meticulous stepwise process. An appropriate cell line of “producer cells” must be chosen and expanded to produce a high enough viral titer; bioreactors are becoming increasingly useful in this field to handle the demand for such a large cell culture. Growing the cells presents the first hurdle, separating the virus from the supernatant is a delicate process and therefore cells are ideally grown serum-free to avoid involving additional components that would complicate purification; conversely, this is obviously not the most efficient method of cell culture. After infection and incubation, a lysis buffer is added followed by a nuclease that degrades nucleic acids of the producer cell. Viral particles are then purified from the lysate through processes that can vary depending on the characteristics of the virus (size, stability under reagents, heat, physical stress, etc.). Methods include centrifugation, ion-exchange chromatography, size exclusion chromatography, tangential flow filtration, and more. Once purified, the virus is tested for contaminants, potency, and identity through methods like PCR or Western blotting (63).

Direct tumor cell cytotoxicity caused by the virus is often a result of the activation of apoptotic pathways, including Ras and caspase signaling. In addition to direct action, a key part of the MOA of an OV is an immune stimulatory effect. This occurs when the presence and/or activity of the virus recruits both adaptive and innate immune responses that might have been otherwise suppressed in the tumor microenvironment. Tumor cell lysis releases TAAs and damage-associated molecular patterns (DAMPs) such as heat shock proteins and uric acid (64). These are recognized by dendritic cells and CD8 + T lymphocytes prompting them to target the tumor and release inflammatory cytokines. Virus-infected tumor cells also express MHC-1 marking them for cell lysis just as in a natural viral infection of healthy cells.

A major hurdle that has arisen in the advancement of OVs has been the balancing act with the body’s immune response. This challenge was mentioned earlier in regards to virus vectors and is even more prevalent in the use of OVs. The virus is at risk of being eliminated by the innate immune response before eliciting any effect on the tumor; consequently, the route of administration is key in this therapy with intratumoral being preferred over IV. The development of an adaptive immune response toward viral antigens also eliminates the possibility of continuous treatment, which is critical in any oncologic therapy. However, the immune memory is at the same time key to eliminating the tumor; use of a parvovirus OV in mice infected with GL261 glioma achieved a complete response that was also immune to rechallenge with xenografted glioma cells, and modified VSV was also shown to induce tumor immunity in B16 melanoma infected mice (64). It is critical therefore that the virus be able to avoid attacking and downregulating host lymphocytes, even though it is possible they would in turn reduce efficacy of the OV. The prime and boost method is utilized yet again with sequential administration of two OVs of different species to circumvent this issue. It is also possible for TAAs released to have an inhibitory effect, the DAMP HMGB1 has shown to increase production of myeloid suppressor cells, which attenuate the immune response (64). The activity of one virus might also not compliment another, the resulting cytokine release of initial therapy could hinder the efficacy of the following OV treatment. This raises the question of how to combine OVs with existing mainstays of cancer therapy, whether they prime or inhibit the immune system.

Currently there are only two OVs that have been brought to market: T-Vec (brand name Imlygic, previously OncoVEX, Amgen) and Oncorine. T-Vec (Amgen) is a modified HSV-1 virus approved by the FDA in 2015 for the treatment of melanoma. It is encoded with a transgene to express GM-CSF; and modified with two gene deletions. The ƴ34.5 gene is responsible for stopping the host cell from shutting down protein synthesis during infection, its deletion makes the virus selective to tumor cells which have mutated to express continual protein synthesis. The α47 gene blocks MHC-1 expression in a natural infection, removal of this gene allows the infected tumor cell to be targeted by the immune system (65). In a phase III trial of metastatic melanoma patients, T-Vec achieved a durable response rate of 16% vs 2% (treatment vs control) when compared against GM-CSF therapy, and 33% vs 0% in another subset. The median OS was also significantly improved by over 4 months (66). An advantage to T-Vec and OV therapy at large is that it is well tolerated with minimal and low grade side effects. The only grade 3 or 4 adverse event seen in the T-Vec treatment arm of the above trial was cellulitis occurring in 2% of patients (65). Oncorine is a modified adenovirus approved in China for head, neck, and esophageal cancer in 2005. It contains a deletion of the E1B gene responsible for inactivating p53 to allow continued viral replication, this makes the virus selective for tumor cells which inactive p53 on their own. A Chinese study published in 2015 compared Oncorine with or without transhepatic arterial chemoembolization therapy in patients with hepatocellular carcinoma. The study showed a significant increase in complete response (28.7% vs 14.8%), OS (12.8 vs 11.6), progression free survival (10.49 vs 9.72), and other parameters (67). Adverse events were similar between the two groups and were low grade and reversible, such as fever, pain, and elevated white blood cells. Naturally occurring OVs without any genetic modification have also shown potential as cancer therapeutics. Reolysin is a wild type strain of reovirus that achieved FDA orphan drug designation in 2015, it targets tumors through its natural selectivity for cells with over-activation of the Ras pathway. A recent phase III trial has shown Reolysin to improve OS in patients with recurrent head and neck cancer when combined with a combination chemotherapy regimen compared to chemotherapy alone (65).

### Bispecific T-Cell Engagers (BiTEs)

**Figure 4:**
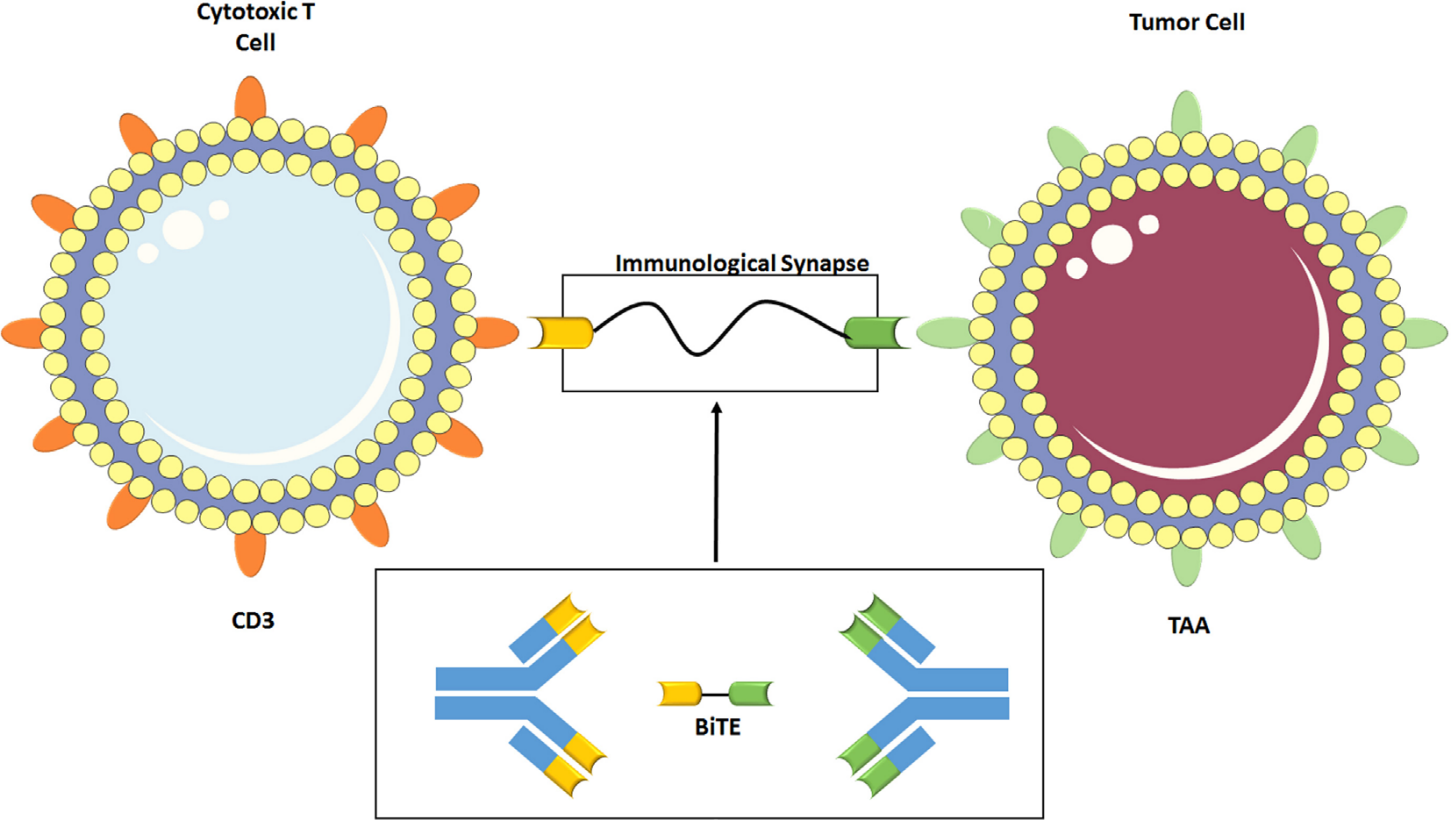
**Construction of a bispecific T-cell engagers (BiTE) is shown in the lower part of the diagram**. The single chain fragment variables (Fab-binding domains) of the two desired antibodies are joined via amino acid linkage. Above, the BiTE binds CD3, a T-cell coreceptor, and the tumor-associated antigen (TAA). The linkage forces the formation of an immunological synapse by drawing the two together, causing recognition of the TAA and activation of the T-cell. Some elements adapted from Servier Medical Art (https://creativecommons.org/licenses/by/3.0/legalcode).

A BiTE is a bispecific antibody featuring the minimal binding domains of the Fab antibody portion (called the single-chain fragment variables) of two antibodies linked via a non-immunogenic, 5-amino acid repetitive linker (68). One binding domain interacts with CD3 on the surface of the T-cell, while the other interacts with the desired TAA. The linkage forces the formation of an immunological synapse (Figure 4), where the T-cell then perforates the tumor cell membrane and releases granzymes that induce a caspase-mediated apoptosis (69), in addition to cytokine release and T-cell proliferation. This process occurs independent of TCR specificity, TCR costimulation, peptide antigen presentation, and without an antibody Fc portion, (68) making it a powerful augment to the body’s natural innate immune response.

Blinatumomab (Amgen) is currently the only approved BiTE therapy, targeting CD19 on B cells, it received FDA accelerated approval in 2014 for the treatment of Philadelphia chromosome–negative relapsed or refractory acute lymphoblastic leukemia (R/R ALL). The trial that served as the basis for approval enrolled 189 R/R ALL patients showing minimum residual disease (MRD, an indicator of poor outcomes) and measured a primary endpoint of CR/CRh (a partial hematologic recovery defined as platelets > 50,000/μl, hemoglobin > 7 g/dl, and absolute neutrophil count > 500/μl) (68). About 43% of patients achieved the primary outcome and MRD was eliminated in 82% of those who responded. OS and RFS were 6.1 and 5.9 months, respectively (70), while not dramatic improvements these outcomes were acceptable considering the drug’s use as salvage therapy in a high-risk and resistant disease. Adverse effects have emerged as an issue in Blinatumomab therapy, about 99% of patients in this trial experienced some grade of adverse effects, often attributable to cytokine release (pyrexia, headache, peripheral edema) or destruction of the B cells (lymphopenia). CRS has been observed in other trials and seems to correlate with disease burden (70), steroid pretreatment with dexamethasone has been identified as an effective manner of controlling CRS. About 68% of patients in the above trial experienced toxicities grade III or above, and about half experienced neurological side effects, identified previously as the dose limiting toxicity, steroid pretreatment can be used to manage these as well. The adverse events observed were all dose dependent and resolved upon the discontinuation of treatment (69). Blinatumomab shows effectiveness at very low clinically achievable doses, the above trial treated patients with 28 µg/day compared to similar conventional antibody therapies which are used in milligram dosages. The drug also features a small protein size at 55 kDa (less than half that of a monoclonal antibody) and rapid clearance, such clearance necessitates a continuous infusion but could also speak for its ability to easily reach the site of action and becomes an asset when needing to quickly reverse toxicities.

Bispecific T-cell engager therapy is currently being expanded to the treatment of solid tumors, under investigation are agents targeting CEA, prostate-specific membrane antigen, and epithelial cell adhesion molecule (EpCAM). Solitomab (AMG110) is an anti-EpCAM BiTE currently in phase I trials. An *in vitro* study using it to treatment uterine and ovarian carcinosarcoma cell lines showed an increase in T-cell cytotoxicity from 1.1% when treated with peripheral blood lymphocytes compared to 19.7% in Solitomab treatment (71).

## Assessing Clinical Outcomes

Monitoring patient response to immune checkpoint inhibitors can in some cases be challenging when treatment results in non-conventional response kinetics, contradictory to those which would be expected in conventional therapies. Responses can vary, some tumors will show the expected immediate response or lack of progression, but others will exhibit a preliminary progression of the tumor that is then followed by responsive or stable disease. This has been called pseudoprogression and occurs as a result of the newly activated T-cells infiltrating the tumor causing what appears radiographically as flaring and progression of the lesion. This necessitates careful timing in the assessment of tumor response to checkpoint inhibitors. For example, it has been recommended that the initial assessments of ipilimumab do not begin until 12 weeks following the start of therapy (15). It also becomes the burden of physicians to differentiate what could be pseudoprogression from what is true tumor progression. As a general rule, pseudoprogression involves new or progressing lesions without any associated response by the patient or worsening of symptoms, in some cases the increased T-cell infiltration can also be confirmed by tumor biopsy.

A set of response criteria specific to I-O therapies were published in 2005 based on the findings by field experts at that time. It is meant to be used as an addendum to the standard response evaluation criteria in solid tumors (RECIST) for assessing therapeutic outcomes in cancer. The four immune-related response criteria (irRC) identified to correlate with positive outcomes are as follows: shrinkage of baseline lesions without new lesions, durable stable disease (followed by a slow, steady decline in total tumor burden in some patients), response after initial increase in total tumor burden, and response in the presence of new lesions (72). The impact of using these adjusted criteria was shown in a study, where the treatment of advanced melanoma patients with Pembrolizumab was evaluated using both irRC and RECIST v1.1; RECIST was found to underestimate treatment benefit in about 15% of patients (73). Novel means of assessment in addition to the new criteria are also still being explored, the unique interaction between I-O therapy and the tumor and its environment make RECIST methods such as measurement of the tumor lesions unreliable. There are currently several clinical trials ongoing evaluating the use of new I-O imaging methods; many include radioligands detectable by PET scanning engineered to target receptors expressed on activated TILs, MDSCs, and checkpoint receptors. ACT therapy offers a unique approach where the genetically modified T-cell is also transfected with a gene that will allow for detection of the marker (72), this is something that could potentially be applied to vaccine therapy as well.

## Conclusion

The currently approved I-O therapies, although few, are already making an impact in the treatment of cancers of many varieties. Their success and the booming interest in this field at large has led to the development of new therapeutics of all types listed above, some of which are summarized in Table 1. This is evidence of the strength and promise of this field, perhaps the next approved I-O therapy could be a long-awaited cancer breakthrough.

## Author Contributions

JK and MC wrote the manuscript, AA and DAA contributed to the writing of the manuscript; SA-O conceived the review and wrote the manuscript.

## Conflict of Interest Statement

The authors declare that the research was conducted in the absence of any commercial or financial relationships that could be construed as a potential conflict of interest.

## Abbreviations

ACT, adoptive cell transfer; ADC, antibody drug conjugate; ADCC, antibody-dependent cell-mediated cytotoxicity; APC, antigen-presenting cell; ARF-1, ADP-ribosylation factor 1; BCC, basal cell carcinoma; BCG, bacille Calmette–Guérin; CAR, chimeric antigen receptor; CCR5, C-C chemokine receptor type 5; CEA, carcinoembryonic antigen; CRPC, castration resistant prostate cancer; CRC, colorectal cancer; CRS, cytokine release syndrome; CSF, colony stimulating factor; CTLA4, cytotoxic T-lymphocyte-associated protein 4; CXCR4, C-X-C chemokine receptor type 4; DAMPs, damage-associated molecular patterns; EpCAM, epithelial cell adhesion molecule; GEMs, glycolipid enriched microdomains; GM-CSF, granulocyte–macrophage colony-stimulating factor; gp100, glycoprotein 100; HSV, herpes simplex viruses; IDO, indoleamine-pyrrole 2,3-dioxygenase; iNOS, inducible nitric oxide synthases; I-O, immuno-oncology; irRC, immune-related response criteria; JNK, c-Jun N-terminal kinase; LPS, lipopolysaccharides; MAC, membrane attack complex; MDSC, myeloid-derived suppressor cells; MRD, minimum residual disease; mTOR, mechanistic target of rapamycin; MVA vaccine, modified vaccinia ankara vaccine; NHL, non-Hodgkin lymphoma; NK, natural killer; NSCLC, non-small cell lung cancer; OVs, oncolytic viruses; PAMPs, pathogen-associated molecular patterns; PI3K, phosphatidylinositol-3-kinases; PD-1, programmed cell death protein 1; PDE5, phosphodiesterase type 5; PP2A, protein phosphatase 2a.; PRRs, patterns recognition receptors; PSMA, prostate-specific membrane antigen; RECIST, response evaluation criteria in solid tumors; R/R ALL, relapsed or refractory acute lymphoblastic leukemia; SCLC, small cell lung cancer; TAAs, tumor-associated antigens; TAMs, tumor-associated macrophages; TCR, T-cell receptor; T_eff_, T-effector cells; TGN, trans Golgi network; TILs, tumor-infiltrating lymphocytes; TLRs, toll-like receptors; T_reg_, T-regulatory cells; TRIM, tripartite motif protein.
